# Rockwall County Medical Society

**Published:** 1892-02

**Authors:** Joe H. Loving

**Affiliations:** Secretary; Fate, Texas


### Society Notes.

### Rockwall County Medical Society.

DEATH OF DR. TAYLOR.—*Resolved*, That the Rockwall County Medical Society deeply regrets the death of our neighbor physician, Dr. R. A. Taylor, of Nevada, Texas.

*Resolved*, That in the death of Dr. Taylor our profession has lost one of its best members, and the public a hard working, honest man. As former President of this Society, he was always ready to do his part for the advancement of the cause of medicine. He was a rare man, a good physician, a kind father, faithful husband, and the highest type of a Christian gentleman.

*Resolved*, That a copy of these resolutions be spread on the minutes, and the Secretary send a copy to his wife, and a copy be sent to DANIEL'S TEXAS MEDICAL JOURNAL for publication.

JOE H. LOVING, Secretary.

Fate, Texas.

